# HPV16 E6 and E7 Upregulate Interferon-Induced Antiviral Response Genes *ISG15* and *IFIT1* in Human Trophoblast Cells

**DOI:** 10.3390/pathogens6030040

**Published:** 2017-09-03

**Authors:** Lea M. M. Ambühl, Annemarie B. Villadsen, Ulrik Baandrup, Karen Dybkær, Suzette Sørensen

**Affiliations:** 1 Center for Clinical Research, North Denmark Regional Hospital/Department of Clinical Medicine, Aalborg University, 9800 Hjørring, Denmark; l.ambuhl@rn.dk (L.M.M.A.); annemarie.j@rn.dk (A.B.V.); utb@rn.dk (U.B.); 2 Department of Hematology, Aalborg University Hospital/Department of Clinical Medicine, Aalborg University, 9000 Aalborg, Denmark; k.dybkaer@rn.dk

**Keywords:** human papillomavirus, trophoblast cell, placenta, interferon, antiviral response

## Abstract

Human papillomavirus (HPV) is suggested to infect trophoblasts in the placenta, and HPV infections are reported to be more prevalent in pregnancies with adverse outcomes. Results are however controversial, and studies investigating the molecular consequences of placental HPV infections are lacking. We studied HPV DNA localization in the placenta in cases of spontaneous abortion/spontaneous preterm delivery as well as in elective abortion/normal full-term delivery. Using in vitro assays, we investigated downstream effects of HPV16 E6 and E7 expression in trophoblast cells at the gene expression level in order to gain increased biological insight into the interaction between HPV and the cellular host. Fluorescent in situ hybridization (FISH), combined with fluorescent immunohistochemistry (FIHC) to target the trophoblast marker CK7 clearly showed, that HPV DNA resides within syncytiotrophoblast cells in the placenta. In vitro HPV16 E6 and E7-transfected trophoblasts were analyzed by RNA sequencing, and results were validated by reverse transcription real time polymerase chain reaction (RT-qPCR) for selected genes in cell lines, as well as in patient material. We show that HPV16 E6 and E7 upregulate interferon-induced antiviral response genes *ISG15* and *IFIT1* in a human trophoblast cell line two-days post-transfection. This is a response that is not observed when assessing the gene expression levels of the same genes in HPV16-positive placenta samples. Investigations on viral activity find that HPV16 E6 and E7 are not transcribed in patients, possibly suggesting that HPV16 syncytiotrophoblast infection may be latent. We conclude that HPV localizes to syncytiotrophoblast cells of the placenta, and that active expression of HPV16 E6 and E7 induce an immediate interferon-induced antiviral response in trophoblast cells, which is not present in HPV-positive placenta samples, suggesting latent infection.

## 1. Introduction

Human papillomavirus (HPV) is a small DNA virus known to cause genital warts and cervical cancer [1]. HPV is described as an exclusively keratinocyte-specific virus [2]. Nevertheless, HPV DNA has been detected in placenta [3,4,5], amniotic fluid [6,7], umbilical cord blood [8] and fetal membranes [9]. Placental HPV infection has been reported to be involved in spontaneous abortion, spontaneous preterm delivery and placental abnormalities [3,4]. Furthermore, it has been suggested that trophoblasts are the predominant target of HPV in the placenta [10], possibly causing abnormal placentation [4,11,12].

HPV E6 and E7 viral proteins are known to be the major transforming proteins in oncogenic HPV [13]. In epithelial cells, HPV E7 binds to the retinoblastoma protein pRb, thereby deregulating the cell cycle, while HPV E6 binds to the tumor suppressor p53 and targets p53 for degradation [14]. This combination allows HPV to override cell cycle checkpoints and to replicate its DNA in non-cycling cells. In vitro studies investigating effects of HPV E6 and E7 on trophoblast cells show that HPV genes mimic their effects in keratinocytes [15]. Physiological changes observed upon HPV infection of trophoblast cells include highly defective endometrial cell recognition, cellular death and induction of a malignant phenotype [12]. Moreover, HPV has been shown to complete its life cycle in various trophoblast cell lines [11,12]. The decrease in trophoblast–endometrial cell adhesion caused by introduction of HPV E6 and E7 may influence the initial step of embryo implantation and suggests potential for abnormal placentation or gestational expulsion of the early embryo [4,12]. However, a convincing role for HPV infection in connection to pregnancy outcome, at the molecular level, as well as effects of HPV on gene expression of trophoblast cells still need to be demonstrated. 

HPV prevalence in pregnant women varies widely and has been reported to depend on study design, tested tissue types, and geographical location of populations analyzed, as well as on used HPV detection methods [16]. Several studies suggest that HPV infection may be linked to spontaneous abortion, spontaneous preterm delivery and placental abnormalities [3,4,17], whereas others are unable to detect HPV in investigated material [18]. In a study performed by our group, HPV prevalence was not significantly different in placental tissue obtained from spontaneous abortions/spontaneous preterm deliveries compared to elective abortions/normal full-term deliveries (Ambühl et al. 2017 [19]). We were previously able to localize HPV DNA in specific placental cells using chromogen in situ hybridization (CISH) and an effect of HPV DNA in placental cells cannot be omitted. Therefore, this study aimed to investigate the cellular target of HPV by co-localization studies and to study downstream effects of HPV transfection in placental trophoblast cells at the gene expression level.

## 2. Results

### 2.1. HPV Localizes in Syncytiotrophoblast Cells of the Placenta

**Figure 1:**
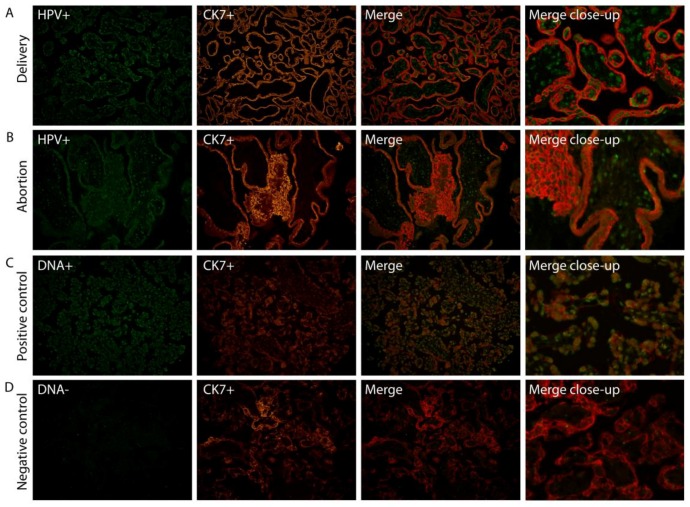
Human papillomavirus (HPV) targets syncytiotrophoblast cells. Combination of HPV-FISH and trophoblast cell FIHC. A representative example of both a normal full-term placenta (**A**) and aborted placental tissue (**B**) is included. HPV signals (HPV+) are in green, trophoblast cell marker (CK7+) in red. A positive control including the DNA(+) probe (**C**) and negative control using the DNA(−) probe (**D**) was used on a control placenta. Magnification: 20×. The merge close-up shows a representative section enlarged 300×. FISH: fluorescent in situ hybridization; FIHC: fluorescent immunohistochemistry; CK7: trophoblast cell marker.

We have previously shown (Ambühl et al. 2017 [19]) using chromogen in situ hybridization (CISH), that HPV DNA appears to be localized to trophoblast cells in placental tissue. To confirm this, we investigated the specific placental cell type infected by HPV by fluorescent co-localization studies enabling a more profound examination of HPV signals in combination with a specific cell marker. HPV DNA localization was determined in placental tissue slides by double staining, combining fluorescent in situ hybridization (FISH) with a fluorescent immunohistochemistry (FIHC) reaction using DNA probes targeting E6/E7 and/or L1 HPV genes, and an antibody against trophoblast cells (CK7). HPV DNA was clearly observed to be localized in syncytiotrophoblast cells (Figure 1).

### 2.2. Gene Expression of JAR Cells is Affected by HPV16 E6 and E7 Expression

**Figure 2:**
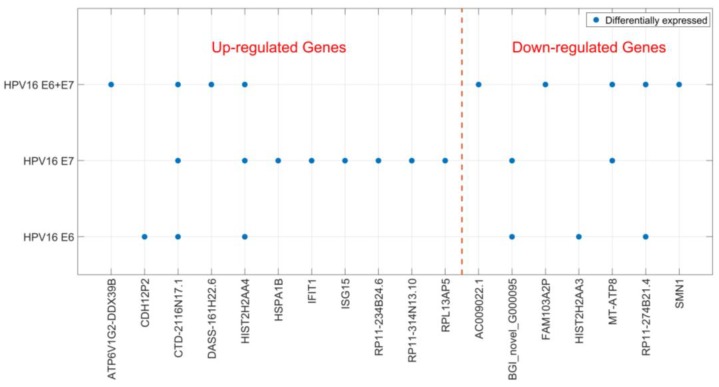
Differentially expressed genes after HPV16 E6- and E7-transfection. Significantly differentially expressed genes (DEGs) after HPV16 E6- and E7-transfection of trophoblast cells are shown depending on the transfected HPV genes. DEGs are divided into up- and down-regulated genes.

Localization of HPV DNA in syncytiotrophoblast cells indicates that HPV may play a biological role in placental function. Therefore, we studied the cellular effects of HPV16 E6 and E7 expression in a trophoblast cell line. We transfected JAR cells (a human choriocarcinoma cell line) with HPV16 E6 and E7-expressing plasmids, both separately and in combination. Using RNA sequencing, transcriptomes of JAR cells transfected with the empty construct were compared to those transfected with HPV genes two-days post-transfection. We found 25 significantly differentially expressed genes (DEGs); 15 genes were upregulated and 10 downregulated (Table 1). The genes that were differentially expressed varied depending on which HPV genes were transfected, and only two genes were found to be differentially expressed in all three transfection experiments (Figure 2). Most DEGs were only detected in one of the transfection conditions and HPV16 E7 appeared to have the greatest impact on the trophoblast transcriptome (Figure 2). Sequencing data were validated for selected genes by reverse transcription real time polymerase chain reaction (RT-qPCR) (for RT-qPCR data see Additional file 2) on transfected JAR cells (Table 2). Altered expression for all tested genes, except for one, was confirmed. Interestingly, two interferon-induced antiviral response genes *ISG15* and *IFIT1* were upregulated after transfection with HPV16 E6 and E7 (Table 2).

*IFIT1*, as well as *ISG15*, are interferon-induced genes reported to be activated immediately and robustly upon viral infection [21,22]. Both genes have previously been studied in relation to HPV [21,23,24,25] and IFIT1 was found to directly interact with HPV L1, leading to the translocation of HPV E1 and inhibition of its DNA helicase activity. Therefore, we decided to investigate if HPV presence in the placenta gives rise to an up-regulation of *IFIT1* and *ISG15* expression in our patient cohort including placental material from spontaneous and elective abortions, as well as from preterm and normal full-term deliveries (Ambühl et al. 2017 [19]). Differential expression of *ISG15* and *IFIT1* was investigated using TaqMan Gene Expression Assays (for RT-qPCR data see Additional file 3) in 32 HPV-positive and 32 HPV-negative placental samples. The data showed that neither *ISG15* nor *IFIT1* were upregulated in HPV-positive placenta samples (Table 3), probably illustrating that HPV was not in its active phase of viral infection at the time when samples were collected from patients.

### 2.3. HPV16 E6 and E7 are not Transcribed in HPV16-Positive Placenta

Simple detection of viral DNA by PCR, hybrid capture assay, or other methods is never equal to a causative role in the adverse outcome of pregnancy or diseases in general. Expression of HPV16 E6 and E7 genes in patient placenta were therefore tested by the use of RT-qPCR to document viral activity (for RT-qPCR data see Additional file 4). Table 4 shows data on gene expression for HPV16 E6 and E7 based on analysis of five HPV16-positive and five HPV-negative placentas. The HPV16-positive cell line SiHa was used as a positive control. Viral activity could only be confirmed for SiHa cells but not for patients with HPV16-positive placentas (Table 4).

## 3. Discussion

Trophoblasts have earlier been claimed to constitute the predominant cellular targets of HPV in placenta by in situ PCR or hybridization [10,26,27]. A direct co-localization between HPV and its target cells has however been lacking. Here we show that HPV is clearly co-localized with syncytiotrophoblast cells of human placentas. 

Placental HPV infection has been suggested to be linked to spontaneous abortion, spontaneous preterm delivery and placental abnormalities [3,4,17]. However, HPV prevalence in pregnant women varies widely and ranges from 0 to 70% in placental tissue [18,28]. Nevertheless, HPV DNA has been co-localized with syncytiotrophoblast cells and therefore potential effects on placental development and consequences for the present pregnancy cannot be excluded. This work aimed therefore at investigating the transcriptome of HPV-transfected trophoblast cells, which allows for an increased insight into the effect on possible deregulation of gene expression. Among others, we identified two up-regulated candidate genes after HPV transfection of trophoblast cells. Both *ISG15*, an interferon-stimulated gene, and *IFIT1*, a gene encoding an interferon-induced protein with tetratricopeptide repeats, are described to be involved in the antiviral response via the interferon system [21,22]. The interferon system constitutes a major role in the host’s innate immune response to viral infections [29]. Viral stress induces transcription of *IFIT1* rapidly and IFIT1 has been described to directly interact with HPV E1 [21]. IFIT1 is reported to translocate E1 from the nucleus to the cytoplasm to inhibit DNA helicase activity of E1 and HPV DNA replication [21]. Contrary to our results, several studies find that HPV down-regulates constitutive expression of interferon-stimulated genes (ISGs) in transcriptome analyses of keratinocytes [23,24,25]. Thus, the cellular target of HPV infection appears to be important for the induced response. IGS15 is an ubiquitin-like protein conjugating to target proteins in the process of ISGylation impairing viral replication in vivo [30,31]. Furthermore, IGS15 is described to induce production of interferon gamma (IFNγ) from T cells, enhance natural killer cell proliferation, and activate monocytes and macrophages via the induced IFNγ [22]. Further investigations are needed to clarify if the immune system of the placenta or the pregnant woman are able to respond to an infection with HPV, thereby possibly preserving the present pregnancy. In addition, others have suggested that the HPV-associated pathological mechanism could include E6 and E7’s effects on the immune system [15,32].

Up-regulation of antiviral genes, *ISG15* and *IFIT1*, was not detected in HPV positive placental tissue. Bearing in mind that the nature of in vitro experiments implies boosting of possible downstream effects due to overexpression and investigation of short-term effects, this might not be surprising. Moreover, placental samples constitute a snapshot of viral infection and gene expression at the time-point of sample collection. HPV infections may have been present for some time. Both *ISG15* and *IFIT1* are found to be induced rapidly and robustly after IFN stimulation upon viral infection [21,33]. Therefore, differences in gene expression may be difficult to measure in patient samples and this illustrates that HPV detected in patients was not in its active phase of viral infection. To test this further, we investigated transcription of HPV16 E6 and E7 in patients with HPV16-positive placentas by RT-qPCR. The impact of viral infections is not directly proportional to detection of viral DNA in clinical samples, as viral activity is crucial for a virus to play a causative role in diseases in general. Viral activity in HPV16-positive patients was not detected. Therefore, we suggest that HPV infections in patients may be latent, as viral DNA is present without any evidence of ongoing viral replication. However, effects of HPV DNA on placental cells cannot be excluded and further investigations on the role of placental HPV infections on pregnancy outcome are needed.

## 4. Materials and Methods 

### 4.1. Placental Material and HPV Screening

Patient material includes placental tissue from the following four study groups: Women who presented with (A) a spontaneous abortion (between week 8 and 22 of gestation); (B) a spontaneous preterm delivery (before 37 + 0 weeks of gestation); (C) an elective abortion (between week 8 and 22 of gestation); and (D) a full-term delivery (≥37 weeks of gestation). All women were recruited at the Departments of Obstetrics and Gynecology at Aarhus University Hospital (preterm deliveries and spontaneous abortions), Aalborg University Hospital (spontaneous abortions), and North Denmark Regional Hospital (full-term deliveries and elective abortions) between March 2014 and April 2016. Women below the age of 18 years of age, with a multifetal pregnancy, known chromosomal or fetal abnormalities, gestational diabetes mellitus, and pre-eclampsia/HELLP (Hemolysis, Elevated Liver enzymes, and Low Platelet count) syndrome were excluded. 

Placental samples were collected in RNAlater and 4% formalin immediately after delivery/abortion, or at the latest after four hours, during which the placenta was stored at 4 °C. Samples in formalin were paraffin-embedded and used for histology analyses and in situ hybridization. Placental tissue stored in RNAlater was used for DNA purification using the QIAGEN AllPrep DNA/RNA Mini purification kit (QIAGEN Inc., Valencia, CA, USA) according to the manufacturer’s instructions. HPV detection was performed by nested PCR using general MY09/11 [34] and GP5+/6+ [35] primers and is described in more detail in Ambühl et al. 2017 [19]. HPV-positive patients in this study are defined as patients where HPV DNA could be detected in the placenta.

### 4.2. Fluorescence in Situ Hybridization (FISH)/Fluorescence Immunohistochemistry (FIHC)

Placenta samples testing positive for HPV by PCR (Ambühl et al. 2017 [19]) were analyzed with Tyramide Signal Amplification (TSA) FISH in combination with FIHC to co-localize HPV in placental cells. Sections measuring 4 µm from formalin-fixed and paraffin-embedded (FFPE) placental samples were used together with the ZytoFast HPV Screening Probe (digoxygenin-labelled) (ZytoVision GmbH, Bremerhaven, Germany), which detects 17 different HPV types (HPV-6, 11, 16, 18, 31, 33, 35, 39, 45, 51, 52, 56, 58, 59, 66, 68, and 82). Histological slides were deparaffinized in xylene and blocked for endogenous peroxidase activity with 0.7% H_2_O_2_ for 15 min. Before the hybridization of probes, tissue slides were pretreated by boiling for 10 min and pepsin digested (ZytoVision GmbH) at 37 °C for 10 min. On all sections 10 μL of DNA probes were applied and covered with coverslips. Slides were denaturated at 75 °C for 5 min and hybridized at 37 °C for 1 h. After stringent washing and protein blocking at 4 °C overnight, probes were visualized using HPR-anti-DIG (PerkinElmer, Waltham, MA, USA) (1:1000) for 30 min, TSA-DIG (PerkinElmer) (1:150) for 5 min, primary antibodies rabbit anti-DIG (1:200, Dako, Carpinteria, CA, USA) and mouse anti-CK7 (1:100, Dako, for trophoblasts) for 30 min, secondary antibodies (Alexa fluor 488 goat anti-rabbit, 1:500 and Alexa fluor 555 goat anti-mouse, 1:200, Dako) for 30 min and DAPI (SantaCruz, Rio Grande City, TX, USA). Each experiment was conducted including the following positive and negative controls: an HPV-infected cervical cancer tissue section applying the HPV screening probe plus TSA, a control placenta applying the ZytoFast DNA(+) Control Probe (ZytoVision GmbH) with and without TSA, and the same control placenta applying the ZytoFast DNA(−) Control Probe (ZytoVision GmbH) plus TSA. The DNA(+) probe detects human Alu repetitive sequences, whereas the DNA(−) probe contains oligonucleotides without known consensus to any naturally occurring sequence.

### 4.3. Cell Culture, Transfection and RNA Extraction

The trophoblast cell line used in this study is JAR (ACC 462, Leibniz-Institut DSMZ GmbH, Braunschweig, Germany). All cells were maintained at 37 °C/5% CO_2_. JAR cells were cultured in RPMI media (biowest, France) supplemented with 10% fetal bovine serum (biowest, Nuaillé, France), and 1% penicillin/streptomycin (biowest, Nuaillé, France).

JAR cells were transiently transfected in 6-well plates (4 × 10^5^ cells per well) using 1 μg DNA and 3:1 FuGENE HD Transfection Reagent (Promega, Madison, WI, USA). The constructs used for transfection were the following: pcDNA3.1_HPV16E6 (insert included sequence of HPV16E6 nt83-nt559 (GenBank K02718.1)) and pcDNA3.1_HPV16E7 (insert included sequence of HPV16E7 nt562-858 (GenBank K02718.1)). Constructs are produced and purchased from GeneArt Gene Synthesis (Invitrogen). Transfection efficiency was assessed by transfecting cells separately with 1 μg pEGFP and was typically 30%. Cells were transfected with pcDNA3.1_HPV16E6 and pcDNA3.1_HPV16E7 separately, and were co-transfected. Furthermore, cells were transfected with the empty pcDNA3.1 vector as a control.

Cells were harvested two days post-transfection and dry cell pellets were stored at −20 °C until RNA extraction. RNA extraction was done using Trizol reagent (Ambion, Life Technologies, Carlsbad, CA, USA) and the mirVana miRNA Isolation Kit (Ambion, Life Technologies). RNA quality was determined by Agilent Bioanalyzer using the Agilent RNA 6000 Nano Kit (Agilent, Santa Clara, CA, USA) according to the manufacturer’s instructions.

### 4.4. Quantitative Reverse Transcription PCR 

RT-qPCR was used for confirmation of HPV16 E6 and E7 transcription after transfection of trophoblast cells. Before complementary DNA (cDNA) synthesis, RNA was treated with DNase I (Invitrogen, Waltham, CA, USA). First, strand cDNA was generated by reverse transcription from 2 μg of total RNA in a total volume of 20 μL using the Affinity Script QPCR cDNA synthesis kit (Agilent, Santa Clara, CA, USA). Quantitative expression of HPV16E6 and HPV16E7 genes was analyzed by real-time PCR using the Stratagene M×3005P system and the Brilliant III Ultra-Fast SYBR green QPCR Master Mix (Agilent, Santa Clara, CA, USA). cDNA was diluted 1:1 with H_2_O and used for real-time PCR. cDNA was amplified in a total PCR reaction volume of 20 μL with specific primers using the following cycle conditions: 95 °C for 10 min, followed by 40 cycles of 95 °C for 30 s, 59 °C for 30 s and 72 °C for 30 s. RT-qPCRs were run in triplicates and No template controls as well as a positive control including cDNA from SiHa cells (HPV16 positive) have been included. Gene expression was normalized using values obtained for the reference gene *GAPDH* (∆Ct = Ct transfected gene-Ct reference gene). Absolute Ct values for HPV16 E6 and E7 confirmed expression of HPV16 E6 and E7 after transfection with pcDNA3.1_HPV16E6 and pcDNA3.1_HPV16E7 (for RT-qPCR data see Additional file 1). A dissociation curve was generated after each experiment to confirm amplification of a single PCR product.

In addition, RT-qPCR was performed to analyze HPV16 E6 and E7 transcription in patient material. Patient RNA for RT-qPCR analysis was purified from placenta samples stored in RNAlater using the QIAGEN AllPrep DNA/RNA Mini purification kit (QIAGEN Inc., Valencia, CA, USA) according to the manufacturer’s instructions. RNA quality was determined by Agilent Bioanalyzer using the Agilent RNA 6000 Nano Kit (Agilent, Santa Clara, CA, USA) according to the manufacturer’s instructions. cDNA synthesis, including DNase I treatment, as well as RT-qPCR, was performed as described above with the following differences: Input for cDNA synthesis was 1.5 μg of total RNA. RT-qPCRs were run in triplicates and No template controls as well as a positive control including cDNA from SiHa cells (HPV16 positive) have been included. *GAPDH* was used as a reference gene. A dissociation curve was generated after each experiment to confirm single PCR product amplification and product specificity (Tm *GAPDH*: 86.6 °C, Tm HPV16 E6: 81 °C , Tm HPV16 E7: 79 °C). Ct-values for specific PCR products are reported (for RT-qPCR data see Additional file 4).

Primers used for amplification were the following:

GAPDH, 5′-GACAGTCAGCCGCATCTTCT-3′, 5′-TTAAAAGCAGCCCTGGTGAC-3′;
HPV16E6, 5′-CTGCAATGTTTCAGGACCCAC-3′, 5′-GTTGTTTGCAGCTCTGTGCAT-3′;
HPV16E7, 5′-AGAACCGGACAGAGCCCATTA-3′, 5′-CGCACAACCGAAGCGTAGA-3′

Validation of candidate genes in HPV transfections of JAR cells and confirmation of in vitro results in patient material (total RNA from placental tissue) were performed by the use of TaqMan Gene Expression Assays (Applied Biosystems) for *IFIT1* (Hs03027069_s1; Cat. # 4331182), *ISG15* (Hs01921425_s1; Cat. # 4331182), *HSPA1B* (Hs01040501_sH; Cat. # 4331182), *SMN1* (Hs02341953; Cat. # 4331182) and *HIST2H2AA4* (Hs00358508_s1; Cat. # 4331182). *GAPDH* (4326317E) and *TBP* (4333769F) were used as endogenous controls. Expression of candidate genes have been validated in three separate transfection experiments and 32 HPV-positive as well as 32 HPV-negative placental samples. RT-qPCRs were run in triplicates and No template controls as well as -RNA controls have been included. cDNA synthesis was done using the High-capacity RNA-to-cDNA kit (Applied Biosystems, Foster City, CA, USA). For validation in cell lines, an input of 100 ng of cDNA was used. Each of the 20-μL RT-qPCR reactions included 10 μL of 2× TaqMan Gene Expression Master Mix and 1 μL of 20× TaqMan Gene Expression Assay (Applied Biosystems). Cycling conditions were: 50 °C for 2 min, 95 °C for 10 min, followed by 40 cycles of 95 °C for 15 s and 60 °C for 1 min. A reference RNA consisting of a mix of equal amounts of RNA from four different HPV-negative placenta samples (one from each study group) plus total RNA from JAR cells was used for generation of standard curves and as an inter-plate calibrator. The quantification of transcripts and calculations on differential expression of candidate genes between JAR cells transfected with HPV16 E6 and E7 or pcDNA3.1 empty vector, as well as between patients with HPV-positive and -negative placentas was reported using the 2(-∆∆Ct) method [36]. The following calculations were performed: ∆∆Ct = ∆Ct HPV vector-∆Ct empty vector and ∆Ct = Ct-transfected gene-Ct reference gene; and ∆∆Ct = ∆Ct HPV-negative patients-∆Ct HPV-positive patients and ∆Ct = Ct gene of interest-Ct reference gene (for RT-qPCR data see Additional files 2 and 3).

### 4.5. RNA Sequencing and DEG Analyses

Total RNA was purified from JAR cells transfected with either: (1) pcDNA3.1_HPV16E6; (2) pcDNA3.1_HPV16E7; (3) pcDNA3.1_HPV16E6 and pcDNA3.1_HPV16E7; or (4) pcDNA3.1 (empty vector). The experiment was performed in triplicates and therefore 12 samples were sent for RNA sequencing at the Beijing Institute of Genomics, Chinese Academy of Sciences (BGI, Beijing, China). Library construction and paired-end transcriptome sequencing (on an Illumina HiSeq 2000/2500 platform, 20M clean reads/sample, 4G clean data) as well as bioinformatics were performed by the BGI using the manufacturer’s protocol. Short sequencing reads were filtered removing reads with adaptors, reads where unknown bases were more than 5% and low-quality reads. Genome mapping of clean reads was performed using HISAT (Hierarchical Indexing for Spliced Alignment of Transcripts) [37]. The NOIseq method described by Tarazona et al. [20] was used for detection of differentially expressed genes. Input parameters were the following: fold-change ≥ 2.0 and probability ≥ 0.8. Raw data from RNA sequencing can be found in the NCBI Sequence Read Archive (SRA, BioProject number PRJNA386626).

### 4.6. Ethical Approval

The local ethical committee (Den Videnskabsetiske Komité for Region Nordjylland) approved this research (N-20130082) on 4 February 2014. Additionally, the project has been reported to the Data Protection Agency. All participants gave informed consent. 

## 5. Conclusions

This study clearly localized HPV to syncytiotrophoblast cells in placenta. Moreover, the study of molecular effects of HPV transfection of trophoblast cells identified two candidate genes, *ISG15* and *IFIT1*, possibly involved in the antiviral response after interferon induction upon viral infection. Further investigations on the role of placental HPV infections are needed and possible consequences for the developing fetus should be considered.

## Figures and Tables

**Table 1:** Differentially expressed genes after RNA sequencing (BGI).

Transfection	Gene ID	Log 2 Fold Change	Probability	Up-/Down-Regulation
HPV16 E6				
	CDH12P2	8.2	0.885014	Up
	CTD-2116N17.1	1.6	0.840587	Up
	BGI_novel_G000095	−1.3	0.833708	Down
	HIST2H2AA3	−4.8	0.953578	Down
	RP11-274B21.4	−7.5	0.810089	Down
	HIST2H2AA4	2.7	0.882313	Up
HPV16 E7				
	CTD-2116N17.1	2.3	0.874182	Up
	RPL13AP5	1.2	0.808273	Up
	RP11-314N13.10	1.1	0.809181	Up
	RP11-234B24.6	7.6	0.832868	Up
	MT-ATP8	−1.6	0.851965	Down
	BGI_novel_G000095	−1.4	0.838509	Down
	IFIT1	6.0	0.817424	Up
	HIST2H2AA4	2.0	0.836634	Up
	ISG15	1.3	0.837189	Up
	HSPA1B	1.3	0.833963	Up
HPV16 E6 + E7				
	CTD-2116N17.1	2.0	0.859646	Up
	MT-ATP8	−3.2	0.916022	Down
	ATP6V1G2-DDX39B	2.1	0.814116	Up
	RP11-274B21.4	−7.5	0.815107	Down
	FAM103A2P	−1.6	0.807606	Down
	AC009022.1	−7.5	0.817934	Down
	DASS-161H22.6	3.8	0.874002	Up
	HIST2H2AA4	2.0	0.837189	Up
	SMN1	−1.1	0.811964	Down

Listed are all the significantly differentially expressed genes found by RNA sequencing of HPV16-transfected JAR cells. Total RNA from transfections in triplicates were sequenced and grouped for comparative analysis using the noisy distribution model NOISeq described by Tarazona et al. 2011 [20]. Cutoff values chosen were the following: a probability *q* = 0.8 and a minimum of 2-fold change compared to the reference transfection using the empty plasmid pcDNA3.1.

**Table 2:** Validation in HPV16-transfected JAR cells.

Gene ID	Transfection	∆∆Ct	Fold-Change (2^−∆∆Ct^)	Validation
**IFIT1**	HPV16 E6	−3.38	10.41	Up-regulation confirmed
	HPV16 E7	−3.72	13.18	
	HPV16 E6 + E7	−2.17	4.5	
**ISG15**	HPV16 E6	−0.75	1.68	Up-regulation confirmed
	HPV16 E7	−0.97	1.96	
	HPV16 E6 + E7	−0.2	1.15	
**HSPA1B**	HPV16 E6	−0.48	1.39	Up-regulation confirmed
	HPV16 E7	−1.21	2.31	
	HPV16 E6 + E7	−0.58	1.49	
**SMN1**	HPV16 E6	0.21	0.86	Down-regulation confirmed
	HPV16 E7	0.48	0.72	
	HPV16 E6 + E7	0.4	0.76	
**HIST2H2AA4**	HPV16 E6	−0.09	1.06	Up-regulation NOT confirmed
	HPV16 E7	−0.06	1.04	
	HPV16 E6 + E7	0.08	0.95	

Differential gene expression found by RNA sequencing are validated in HPV16-transfected JAR cells for the selected genes *IFIT1, ISG15, HSPA1B, HIST2H2AA4, SMN1*. ∆∆Ct summarizes Ct values for three transfection experiments and RT-qPCR was run in triplicates. All Ct values were normalized against the reference gene *GAPDH*.

**Table 3:** Validation in patient samples.

Gene ID	∆∆Ct	Fold Change (2^−∆∆Ct^)	Number of Samples Tested/Included
IFIT1	0.065	0.96	32/16 HPV positives
			32/14 HPV negatives
ISG15	0.143	0.91	32/18 HPV positives
			32/16 HPV negatives

Differential expression of IFIT1 and ISG15 was evaluated in placental RNA from 32 HPV-positive and 32 HPV-negative placental samples. Number of samples included refers to samples with valid RT-qPCR results according to standard curves and dynamic range. The fold change for HPV-positive vs. negative placental samples is included. Ct values were normalized against reference genes *GAPDH* and *TBP*.

**Table 4:** Viral activity in placental tissue.

Sample	Ct GAPDH	∆Ct HPV16 E6	∆Ct HPV16 E7
SiHa	13.83	5.32	4.84
HPV+ patient 1	16.2	-	-
HPV+ patient 2	15.36	-	-
HPV+ patient 3	14.92	-	-
HPV+ patient 4	17.39	-	-
HPV+ patient 5	15.26	-	-
HPV− patient 1	16.31	-	-
HPV− patient 2	16.61	-	-
HPV− patient 3	14.2	-	-
HPV− patient 4	14.7	-	-
HPV− patient 5	14.86	-	-

Expression of HPV16 E6 and E7 were evaluated in placental RNA from five patients with HPV16-positive and five patients with HPV-negative placentas by RT-qPCR. The HPV16 positive cell line SiHa was used as a positive control. Ct-values for specific PCR products are reported. *GAPDH* was used as a reference gene.

## Supplementary Materials

The following are available online at www.mdpi.com/2076-0817/6/3/40/s1, Additional files 1–4. The RNA sequencing datasets produced and analyzed in this study are available in the NCBI Sequence Read Archive (SRA), https://www.ncbi.nlm.nih.gov/sra. The BioProject number is PRJNA386626.
